# Comparing Prognostic Factors of Cancers Identified by Artificial Intelligence (AI) and Human Readers in Breast Cancer Screening

**DOI:** 10.3390/cancers15123069

**Published:** 2023-06-06

**Authors:** Cary J. G. Oberije, Nisha Sharma, Jonathan J. James, Annie Y. Ng, Jonathan Nash, Peter D. Kecskemethy

**Affiliations:** 1 Kheiron Medical Technologies, 112-116 Old St., London EC1V 9BG, UK; 2 Breast Screening Unit, Leeds Teaching Hospital NHS Trust, Leeds LS14 6UH, UK; 3 Nottingham Breast Institute, City Hospital, Nottingham University Hospitals NHS Trust, Nottingham NG5 1PB, UK

**Keywords:** breast cancer, AI, population screening, cancer prognosis, cancer survival

## Abstract

**Simple Summary:**

Current AI algorithms show breast cancer detection rates that are comparable to human readers, but it is not clear whether AI and humans detect cancers with similar characteristics. As these factors will influence survival, we aimed to compare the invasiveness status, histological grade, lymph node stage, and tumour size of cancers. Women diagnosed with breast cancer between 2009 and 2019 from three UK double-reading sites were included in this retrospective cohort evaluation. From 1718 screen-detected cancers (SDCs) and 293 interval cancers (ICs), AI indicated 85.9% and 31.7%, respectively, as suspicious. The first human reader detected 90.8% of SDCs and 7.2% of ICs. There were no differences in the detected proportion for any of the investigated prognostic factors. The AI algorithm detected more ICs. These findings imply that using AI has limited or no downstream effects on screening programmes, supporting its potential role in the double-reading workflow.

**Abstract:**

Invasiveness status, histological grade, lymph node stage, and tumour size are important prognostic factors for breast cancer survival. This evaluation aims to compare these features for cancers detected by AI and human readers using digital mammography. Women diagnosed with breast cancer between 2009 and 2019 from three UK double-reading sites were included in this retrospective cohort evaluation. Differences in prognostic features of cancers detected by AI and the first human reader (R1) were assessed using chi-square tests, with significance at *p* < 0.05. From 1718 screen-detected cancers (SDCs) and 293 interval cancers (ICs), AI flagged 85.9% and 31.7%, respectively. R1 detected 90.8% of SDCs and 7.2% of ICs. Of the screen-detected cancers detected by the AI, 82.5% had an invasive component, compared to 81.1% for R1 (*p*-0.374). For the ICs, this was 91.5% and 93.8% for AI and R1, respectively (*p* = 0.829). For the invasive tumours, no differences were found for histological grade, tumour size, or lymph node stage. The AI detected more ICs. In summary, no differences in prognostic factors were found comparing SDC and ICs identified by AI or human readers. These findings support a potential role for AI in the double-reading workflow.

## 1. Introduction

The positive effect of breast cancer screening on survival rates is mainly due to the early detection of invasive cancers [1,2]. Recently, the relative sensitivity of human readers for grade 3 invasive cancers compared to grade 1 invasive cancers was estimated as low as 52% [3]. Although this indicates that there might be room for improvement, it is even more important to ensure that there is no decrease in the detection of clinically important diseases when introducing new workflows or new technologies.

Studies have reported that AI performance is comparable to or might even outperform humans in the interpretation of screening studies [4,5,6,7,8]. In these studies, performance is often assessed in terms of general outcome metrics such as cancer detection rate and recall rate [9], but information on the prognostic features of screen-detected breast cancers is frequently not provided. However, patient outcomes, including survival, are dependent on the characteristics of the detected tumours [10,11]. There are two clinical issues of importance: the first is to ensure that AI detects aggressive, potentially fatal cancers early; the second is to check whether AI detects an excess of in situ or otherwise “minimal” cancers, in which case it might be contributing to overdiagnosis. The need to assess the downstream effects of using AI is also emphasised by the UK National Screening Committee, which recently published their approach for reviewing AI evidence [9]. 

While the topic is considered important, original research on it is scarce. Lee et al. reported that the AI gave higher malignancy scores to invasive cancers compared to non-invasive cancers but did not compare the proportion found by the AI to that of human readers [12]. McKinney et al. reported in a supplementary file that there were no differences in characteristics of tumours detected by AI or by human readers, but on a limited set of screen-detected cancers [13].

The American Joint Committee on Cancer now incorporates biomarkers such as hormone receptor status and HER2 status into the 8th edition of its cancer staging system [14], but the traditional prognostic features of tumour size, lymph node involvement, and histological grade are still widely and consistently used to assist in therapeutic decision-making as they confer strong, independent associations with breast cancer survival [15,16]. A comparison of AI and human performance for subgroups based on invasive/in situ status of disease, histological grade, lymph node stage, and tumour size would provide useful information for the way that AI could be integrated in screening programmes in order to support sustainability by presenting opportunities to save workload while maintaining or even improving screening performance. The aim of this analysis was to compare the characteristics of cancers detected by artificial intelligence (AI) and human readers using these important prognostic features. 

## 2. Materials and Methods

### 2.1. Study Populations and Samples

A retrospective study was carried out at four centres: three in the UK and one in Hungary. The trial was registered at ISRCTN (https://www.isrctn.com (accessed on 15 March 2023), trial registration number ISRCTN18056078). The current analysis is based on data from the UK sites only, which all adhere to a three-year screening interval. The study population consisted of women who participated in breast cancer screening and were diagnosed with breast cancer. The study period ranged from 2009 to 2019. All cancers detected in this consecutive ten-year historical cohort of de-identified cases were included. The three UK centres included Leeds Teaching Hospital NHS Trust (LTHT), Nottingham University Hospitals NHS Trust (NUH), and United Lincolnshire Hospitals NHS Trust (ULH). Details of the cohort have been published elsewhere [17].

Mammograms were acquired on equipment from a single vendor at each screening site (Hologic at LTHT, GE Healthcare at NUH, and Siemens at ULH). In the UK, women are routinely invited between the ages of 50 and 70, with women over 70 years of age able to self-refer. The screening programmes used double human reading as the standard of care throughout the study period. Discrepancies between readers in the double-reading workflow were resolved by arbitration using an independent third reader or consensus, depending on local screening centre protocols. When readers agreed to recall a case, either a “recall” decision was reached or an arbitration performed by a single or group of radiologists made the definitive “recall” or “no recall” decision, also depending on the local screening centre protocols. For the current analysis, human reader performance was based on the first reader’s opinion (R1). At all sites, the historical first reader’s opinion was made in isolation, and the second reader had access, at their discretion, to the opinion of the first. Therefore, the first human reader’s opinion is a more reliable indicator of individual human performance.

### 2.2. AI System

All study cases were analysed by the Mia^TM^ version 2.0 ‘AI system’, developed by Kheiron Medical Technologies. The AI system works with standard DICOM (Digital Imaging and Communications in Medicine) cases as inputs, analyses four images with two standard full field digital mammography (FFDM) views per breast (i.e., CC and MLO), and generates a binary suggestion of “recall” (for further assessment due to suspected malignancy) or “no recall” (until the next screening interval). The AI system’s output is deterministic and is based on a single prediction per case. The system used pre-defined thresholds for “recall” or “no recall”.

The AI software version was fixed prior to the study. All study data came from participants, whose data were never used in any aspect of algorithm development. 

### 2.3. Data Collection

All positive cases, both screen-detected and interval cancers, were pathology proven malignancies. The presence of an invasive component, histological grade, tumour size, and lymph node status were retrieved from the NHS National Breast Screening Service (NBSS) database, including cancer registry information. In addition, the human readers’ recall decisions were also retrieved from the NBSS database.

### 2.4. Statistical Analysis

The screen-detected cancers and interval cancers (IC) were analysed separately. For all tumour characteristics, numbers, percentages, and 95% Wilson score confidence intervals were reported [18,19]. The proportion detected by the AI system was compared to R1 using the chi-square test. If subcategories contained too few cases, Fisher‘s exact test was applied. Statistically significant differences in the detection of cancers with specific prognostic factors were concluded if the chi-square test resulted in a *p*-value of <0.05. Invasive tumour size was classified as ≤20 mm or >20 mm in accordance with the TNM staging system. In addition, a refined categorisation into 5 categories was analysed: ≤5 mm, 5–10 mm, >10–20 mm, >20–50 mm, and >50 mm. 

The sensitivity of AI and R1 for each prognostic subgroup was compared using McNemar’s test [20]. Point estimates and 95% confidence intervals for sensitivity were reported. The relative sensitivity of the AI, defined as the percentage of cancers that the AI detected from the group of R1-detected cancers, and the relative sensitivity of R1, defined as the percentage of cancers that R1 detected from the group of AI-detected cancers, were also reported.

Commonly used outcome metrics for breast screening programmes have recently been reported for the study population [17]. These metrics include recall rate (RR; number of cases suggested to call back for further assessment), cancer detection rate (CDR; number of cancers detected per 1000 screens), sensitivity (SEN), specificity (SPEC), and positive predictive value (PPV; number of cancers detected divided by the number of cases suggested to call back for further assessment). The confidence intervals for the sensitivity in the prognostic subgroups, the relative sensitivity, and the screening outcome metrics are based on 4000 bootstraps. All analyses were performed using Python 3.8 [21].

## 3. Results

### 3.1. Characteristics of the Study Sample

**Figure 1:**
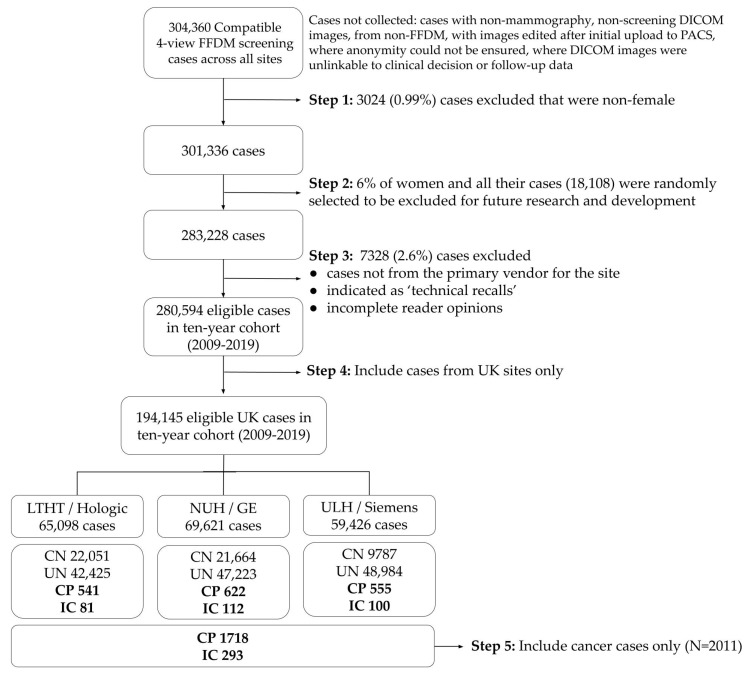
Flow chart of participants. The cases are shown per screening centre/vendor. CN—confirmed negatives; UN—unconfirmed; CP—screen-detected cancer; IC—interval cancer.

Data from a previously performed study was used [17]. For the current analysis, we focused on the patients diagnosed with cancer. In addition, we only included the UK population, as this data was more complete. While the original study excluded patients with a history of breast cancer, they were included in this analysis. This resulted in a study sample of 194,145 UK cases. Figure 1 shows the study flow diagram. There were 1718 screen-detected cancers and 293 interval cancers among 194,145 screening examinations.

### 3.2. General Performance Metrics

**Figure 2:**
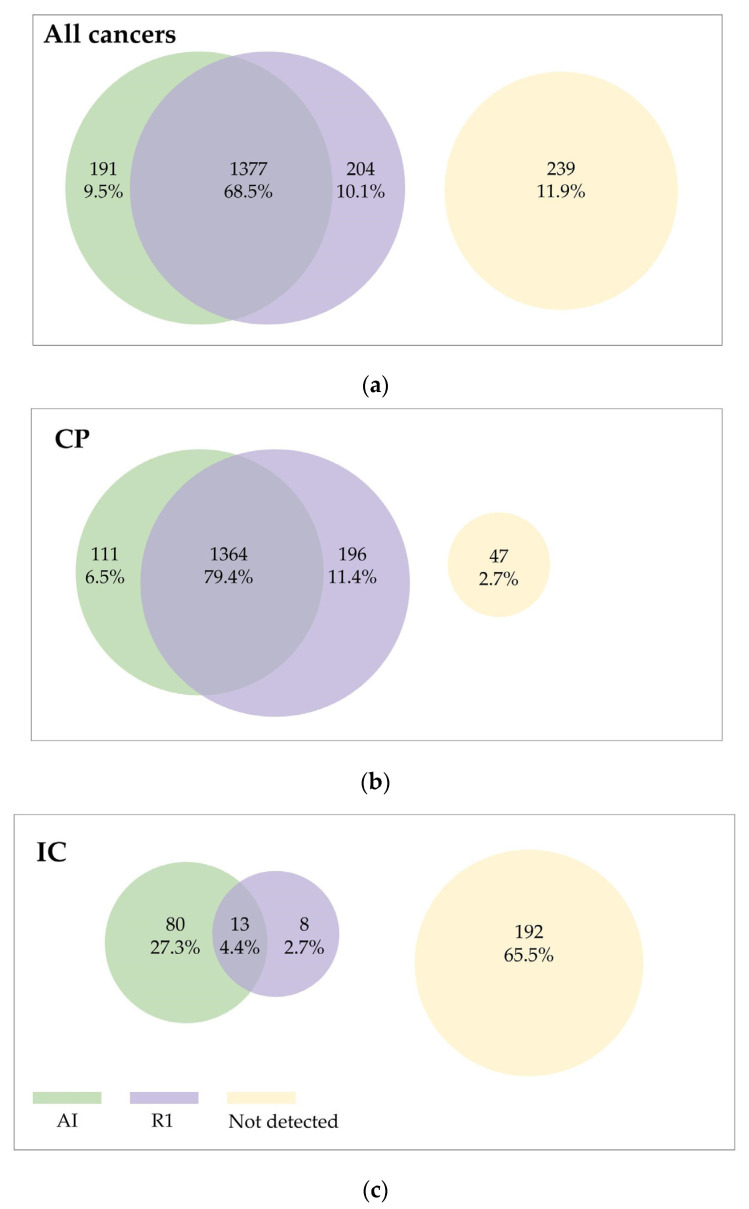
Venn diagram showing cancer detection for the first human reader (R1, purple circle) and AI (green circle). The overlap indicates the cancers being detected by both R1 and the AI. The yellow circle indicates the cancers that were not detected by R1 or the AI. Cancer detection is shown for (**a**) all cancers, (**b**) CP (screen-detected cancers), and (**c**) IC (interval cancers).

The AI system detected 85.9% (1475) of the screen-detected cancers and indicated 31.7% (93) of the original screening mammograms of ICs as suspicious. R1 detected 90.8% (1560) of the screen-detected cancers and 7.2% (21) of the original screening mammograms of ICs (Table S1, Figure 2). General outcome metrics for the initial study have been reported elsewhere [17].

### 3.3. Distribution of Tumour Characteristics

#### 3.3.1. Invasive Cancer Detection

**Figure 3:**
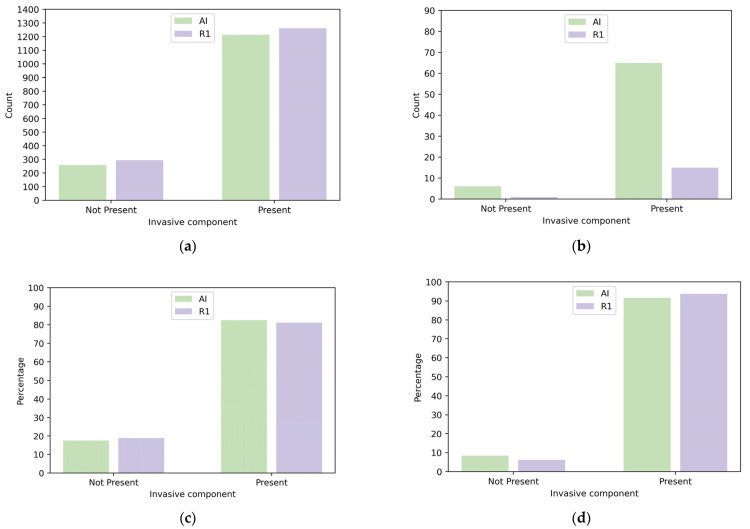
Cancers with an invasive component detected by AI versus Reader 1: (**a**) number of screen-detected cancers; (**b**) number of interval cancers; (**c**) percentage of screen-detected cancers; and (**d**) percentage of interval cancers.

The screen-detected cancers were invasive in 82.5% (95% CI 80.4%–84.3%) of the cases for the AI, while this was 81.1% (95% CI 79.1%–83.0%) for R1, resulting in a non-significant *p*-value of 0.374 (Table 1, Figure 3). For the ICs detected by the AI, a proportion of 91.5% (95% CI 82.8%–96.1%) was invasive, while this was 93.8% (95% CI 71.7%–98.9%) for R1 (*p* = 0.999; Table 1, Figure 3).

#### 3.3.2. Histological Grade

The distribution of the tumour characteristics for the invasive cancers is shown in Table 2 and Table 3, for the screen-detected cancers and ICs, respectively, as well as Figure 3a–d. A refined categorisation for whole tumour size and invasive tumour size can be found in Table S1. The 95% confidence intervals for the histological grade distribution showed large overlap, for screen-detected as well as ICs. The screen-detected invasive cancers consisted of 17.2% and 17.3% of grade 3 tumours, for the AI and R1, respectively (*p* = 0.981). For the invasive ICs, the percentage of grade 3 cancers was 35.9% for the AI and 33.3% for R1 (*p* = 0.851). The absolute number of detected ICs was much lower than the number of screen-detected invasive cancers, resulting in wider confidence intervals (Table 3).

#### 3.3.3. Tumour Size

There were no differences in invasive tumour size for screen-detected cancers identified by AI or R1: 83.1% (907/1091) and 83.1% (941/1133) were ≤20 mm, respectively, *p* = 0.995. A comparison of the whole tumour size, which includes the size of invasive and non-invasive disease, showed no difference between AI and R1: 66.9% (723/1080) and 66.1% (742/1122). For the invasive tumour size of the ICs, the proportion of small cancers for the AI (56.7%) was lower than for R1 (64.3%), but the chi-square test resulted in a non-significant *p*-value of 0.826 (Table 3). For invasive as well as whole tumour size, the 95% confidence intervals largely overlapped. Results for a refined categorisation into 5 categories (≤5 mm, 5–10 mm, 10–20 mm, 20–50 mm, and >50 mm) can be found in Supplementary Table S2.

#### 3.3.4. Lymph Node Stage

The proportion of screen-detected cancers with a positive lymph node status was 20.0% and 20.1% for the AI and R1, respectively (*p* = 0.959), while these proportions were 36.4% and 40.0% for the ICs (*p* = 0.999). Additionally, for this tumour characteristic, the overlap of the 95% confidence intervals was large.

### 3.4. Sensitivity and Relative Sensitivity per Prognostic Subgroup of R1 and AI

The sensitivity of R1 and AI for cancers with specific characteristics is shown in Table 4. While R1 has a significantly higher sensitivity for non-invasive cancers, the AI shows a somewhat higher sensitivity for cancers with a large invasive tumour size and for high-grade tumours. This finding is mainly due to the higher proportion of ICs that the AI detects, as shown in Table S3a,b for screen-detected and interval cancer, respectively. The 95% confidence intervals show again a large overlap for most characteristics, except for non-invasive cancers, where R1′s sensitivity is higher than the AI. Table S4 displays the relative sensitivity per prognostic subgroup. For most subgroups, the relative sensitivities of the AI and R1 are in line with each other, indicating that the number of cancers detected by R1 from the group of cancers detected by the AI is comparable to the number of cancers detected by the AI from the group of cancers detected by R1.

## 4. Discussion

The aim of this large-scale evaluation was to compare the prognostic features of cancers detected by AI and human readers. The proportion of invasive cancers detected by AI was comparable to the proportion detected by R1. Additionally, for the tumour characteristics of the invasive cancers, no differences were found between the AI and R1. Results for screen-detected cancers and ICs were similar, although the number of ICs in the dataset was lower, which resulted in broader confidence intervals. In addition, the AI detected a higher number of ICs than the human readers (31.7% versus 7.2%, respectively), indicating the complementary value of the AI to humans. Our results indicate that using AI in breast screening will not have downstream effects on screening programmes. Moreover, the findings suggest that cancers detected by AI and human readers are likely to have a similar clinical course and outcome, supporting the potential role of AI as a reader in the double-reading workflow. As such, these results pave the way for prospective assessment of AI, either in clinical studies or in service evaluations, as a safe next step.

Very few previous studies have assessed the prognostic features of cancers detected by AI. McKinney et al. compared the histological features of cancers detected by the first human reader and the AI system and found no difference in the presence of invasive disease, histological grade, or size, but their analysis only included 414 cancers [13]. By contrast, our analysis is based on 1718 screen-detected cancers and 293 interval cancers. Lee et al. reported that the AI gave higher malignancy scores to invasive cancers compared to non-invasive cancers but did not compare the proportion found by the AI to that of human readers [12]. Leibig et al. reported the sensitivity of a large number of screen-detected cancers for AI versus radiologists, but the operating point was not pre-specified, making it difficult to interpret their results in terms of real-world performance [22].

The study of Leibig et al. only included screen-detected cancers, while we analysed screen-detected as well as interval cancers [22]. This gives a more complete picture of the cancers that can be detected with AI. We have shown that the sensitivity for prognostic subgroups of R1 and AI is comparable, although AI had a somewhat higher sensitivity for cancers with a larger invasive tumour size and a higher grade, while R1 was more sensitive in finding non-invasive cancers. These differences relate to the fact that the AI detected more ICs than R1.

It is well known that interval cancers have less favourable prognostic features compared to screen-detected cancers [23]. Detecting these cancers earlier has the potential to reduce breast cancer mortality. In our study, AI was able to detect 31.7% of screens that subsequently presented as interval cancers, and it is reassuring to note that grade 3 tumours were overrepresented within the group of interval cancers detected by AI, with 35.9% being histological grade 3 compared to 17.2% of screen-detected cancers flagged by AI. Our findings are very similar to those of Larsen et al., who in a recent large retrospective study found that an AI system set at a threshold to mirror the average human reader’s rate of positive interpretations was able to flag 30.7% of interval cancers, with 33% being grade 3 [5]. In both studies, the absolute number of interval cancers is low compared to the number of screen-detected cancers, so confidence intervals are wider and differences are not statistically significant, but the ability of an AI algorithm to identify clinically significant cancers sooner would be beneficial to breast cancer screening programmes.

In the last decades, there has been an ongoing debate about the benefits and harms of breast cancer screening in terms of overdiagnosis, whether of ductal carcinoma in situ (DCIS) or invasive cancer [24,25,26,27]. It is reassuring that in our study, the AI detected a similar pattern of cancers as the human reader. This implies that expected changes in the downstream impact, in terms of clinical outcomes as well as health economics, will be minimal if AI is used as an independent reader. As such, these expected minor changes strengthen our opinion that it is safe to initiate prospective studies and service evaluations where AI is actually integrated into the breast screening workflow. At the moment, these studies and evaluations are lacking [28,29,30].

The strength of our study is that the data has been acquired from large real-world screening populations with mammograms acquired from multiple mammography equipment vendors. However, the missing data for some cancer cases might be seen as a limitation. In addition, cancers were detected several months to three years after the initial screening. This delay makes it difficult to interpret the tumour characteristics, as it is unknown whether a biopsy and subsequent surgery performed at the time of the initial screening would have resulted in a smaller or non-invasive cancer. In some cases, cancer might not have been present at all on the initial screen. Additionally, interval cancer data is incomplete for the later part of the ten-year screening period as not enough time has elapsed for all interval cancer cases to have presented or been notified. As the AI system has been shown to be more sensitive than R1 on interval cancers, it is expected that with a more complete collection of interval cancers, the relative sensitivity of the AI system to R1 would be revealed to be greater. For the same cohort and AI system as in this study, this was reported to be the case where the relative difference in sensitivity of the AI system to R1 ranged from −1.4 to 0.8% across UK sites for the whole study time period, including the later years with missing interval cancer data, but was revealed to be greater, ranging from 2.5% to 7.6% for a study time period with more complete interval cancer data [17]. Finally, integrating AI into the screening workflow can be carried out in multiple ways, and the interplay between human readers and AI is complex. Ultimately, prospective data will be needed to determine the detected cancer spectrum of a breast screening programme where AI has been implemented.

Despite these limitations, our study, based on a large number of screen-detected breast cancers and interval cancers, provides valuable insights into the cancer spectrum detected by AI compared to human readers. The ability of AI to detect breast cancers with similar prognostic features to human readers suggests that cancers detected by AI and human readers will have a comparable clinical course and outcome, including survival. In addition, AI also has the potential to identify interval cancers earlier, which is likely to be of benefit to individual women and the wider screening programme. Prospective studies combining an AI opinion and a human reader are currently lacking and will be needed to better understand the effect on cancer detection, interval cancer rates, and prognostic features.

## 5. Conclusions

The results of this analysis are based on a large number of screen-detected and interval cancers. The AI showed that it can potentially detect interval cancers earlier, which could be beneficial for individual women and the wider screening programme. The cancers detected by the AI and by human readers were comparable in terms of invasiveness, histological grade, tumour size, and lymph node status. Therefore, cancers detected by AI are expected to have a similar clinical course and outcome, including survival. This implies that using AI in a double-reading workflow will have no or limited effects on screening programmes.

These findings indicate that it is safe to integrate AI into breast cancer screening programmes. As such, it paves the way for initiating prospective studies and service evaluations.

## Figures and Tables

**Table 1:** Presence of an invasive component in the detected cancers.

	**Screen Detected Cancers**							
	**Detected by R1**			**Detected By AI**			**Total**	
	**N = 1560**	**%**	**95% CI**	**N = 1475**	**%**	**95% CI**	**N = 1718**	*p* *
Invasive component								
present	1261	81.1%	79.1–83.0%	1213	82.5%	80.4–84.3%	1385	0.374
not present	293	18.9%	17.0–20.9%	258	17.5%	15.7–19.6%	326	
missing	6			4			7	
	**Interval Cancers**							
	**Detected by R1**			**Detected by AI**			**Total**	
	**N = 21**	**%**	**95% CI**	**N = 93**	**%**	**95% CI**	**N = 293**	*p* **
Invasive component								
present	15	93.8%	71.7–98.9%	65	91.5%	82.8–96.1%	222	0.999
not present	1	6.3%	1.1–28.3%	6	8.5%	3.9–17.2%	15	
missing	5			22			56	

Abbreviations: R1—human reader 1; AI—artificial intelligence. * *p*-values are based on the chi-square test. ** *p*-values are based on the Fisher exact test.

**Table 2:** Characteristics of invasive cancers detected on screen by a human reader or AI.

		Detected by R1			Detected by AI			
		N = 1261	%	95% CI	N = 1213	%	95% CI	*p* *
Histological grade	1	329	27.1%	24.7–29.7%	313	26.9%	24.4–29.5%	0.981
	2	673	55.5%	52.7–58.3%	651	55.9%	53.1–58.8%	
	3	210	17.3%	15.3–19.6%	200	17.2%	15.1–19.5%	
	Missing	49			97			
Whole tumour size	≤20 mm	742	66.1%	63.3–68.8%	723	66.9%	64.1–69.7%	0.720
	>20 mm	380	33.9%	31.2–36.7%	357	33.1%	30.3–35.9%	
	Missing	139			181			
Invasive tumour size	≤20 mm	941	83.1%	80.8–85.1%	907	83.1%	80.8–85.2%	0.995
	>20 mm	192	16.9%	14.9–19.2%	184	16.9%	14.8–19.2%	
	Missing	128			170			
Lymph node status	Negative	912	79.9%	77.4–82.1%	878	80.0%	77.6–82.3%	0.959
	Positive	230	20.1%	17.9–22.6%	219	20.0%	17.7–22.4%	
	Missing	119			164			

Abbreviations: R1—Human reader 1; AI—artificial intelligence; CI—confidence interval. * *p*-values are based on a chi-square test.

**Table 3:** Characteristics of invasive interval cancers detected by human reader or AI.

		Detected by R1			Detected by AI			
		N = 15	%	95% CI	N = 65	%	95% CI	*p* *
Histological grade	1	3	20.0%	7.0–45.2%	16	25.0%	16.0–36.8%	0.999
	2	7	46.7%	24.8–69.9%	25	39.1%	28.1–51.3%	
	3	5	33.3%	15.2–58.3%	23	35.9%	25.3–48.2%	
	Missing	0			1			
Whole tumour size	≤20 mm	3	37.5%	13.7–69.4%	19	44.2%	30.4–58.9%	0.999
	>20 mm	5	62.5%	30.6–86.3%	24	55.8%	41.1–69.6%	
	Missing	7			22			
Invasive tumour size	≤20 mm	9	64.3%	38.8–83.7%	34	56.7%	44.1–68.4%	0.766
	>20 mm	5	35.7%	16.3–61.2%	26	43.3%	31.6–55.9%	
	Missing	1			5			
Lymph node status	Negative	6	60.0%	31.3–83.2%	28	63.6%	48.9–76.2%	0.999
	Positive	4	40.0%	16.8–68.7%	16	36.4%	23.8–51.1%	
	Missing	5			21			

Abbreviations: R1—Human reader 1; AI—artificial intelligence; CI—confidence interval. * *p*-values are based on the Fisher exact test.

**Table 4:** Sensitivity per prognostic subgroup for screen-detected and interval cancers combined.

Variable		N	Detected AI	SEN AI	95% CI	Detected R1	SEN R1	95% CI	*p*
Invasive Component	not present	341	264	77.4%	72.9–81.8%	294	86.2%	82.5–89.9%	<0.001
	present	1607	1278	79.5%	77.6–81.5%	1276	79.4%	77.4–81.4%	0.954
	missing	63							
Tumour grade	grade 1	401	329	82.0%	78.3–85.6%	332	82.8%	78.9–86.5%	0.836
	grade 2	845	676	80.0%	77.2–82.6%	680	80.5%	77.7–83.0%	0.805
	grade 3	307	223	72.6%	67.6–77.5%	215	70.0%	64.9–75.3%	0.322
	missing	54							
Whole tumour size	≤20 mm	911	742	81.4%	78.9–83.9%	745	81.8%	79.2–84.2%	0.880
	>20 mm	492	381	77.4%	73.7–81.1%	385	78.3%	74.6–81.9%	0.752
	missing	204							
Invasive tumour size	≤20 mm	1168	941	80.6%	78.3–82.7%	950	81.3%	79.1–83.5%	0.594
	>20 mm	289	210	72.7%	67.5–77.8%	197	68.2%	62.8–73.5%	0.092
	missing	150							
Lymph node status	negative	1110	906	81.6%	79.3–83.8%	918	82.7%	80.4–84.9%	0.460
	positive	298	235	78.9%	74.2–83.6%	234	78.5%	73.7–83.1%	0.999
	missing	199							

Abbreviations: AI—artificial intelligence; R1—human reader 1; CI—confidence interval. Tumour grade, whole tumour size, invasive tumour size, and lymph node status were only assessed for cancers that had an invasive component present. *p* values are based on McNemar’s test.

## Data Availability

Restrictions apply to the availability of these data. Data generated or analysed during the study are available from the corresponding author by request. The imaging datasets are obtained under licences for this study and are not publicly available.

## Supplementary Materials

The following supporting information can be downloaded at: https://www.mdpi.com/article/10.3390/cancers15123069/s1: Table S1. Agreement between human reader and AI for detection of cancers; Table S2a. Size of invasive cancers detected on screen by human reader or AI; Table S2b. Size of invasive interval cancers detected by human reader or AI; Table S3a. Sensitivity per prognostic subgroup for screen-detected cancers; Table S3b. Sensitivity per prognostic subgroup for interval cancers; Table S4. Relative Sensitivity of AI and R1 per subgroup for screen-detected and interval cancers combined; Figure S1a. Barplot Histological grade; Figure S1b. Barplot whole tumour size; Figure S1c. Barplot invasive tumour size; Figure S1d. Barplot lymph node stage.
