# Supplementary material for: Comparing Prognostic Factors of Cancers Identified by Artificial Intelligence (AI) and Human Readers in Breast Cancer Screening
Source: Cancers (Basel). 2023 Jun 6;15(12):3069. doi: 10.3390/cancers15123069 (PMC10296295; doi:10.3390/cancers15123069)
Supplement: Supplementary file 1 [file cancers-15-03069-s001.zip › cancers-2340101-supplementary.pdf]

Supplementary Materials

Comparing prognostic factors of cancers identified by Artificial Intelligence (AI) and human readers in breast cancer screening

Cary J.G. Oberije, Nisha Sharma, Jonathan James, Annie Y. Ng, Jonathan Nash and Peter D. Kecskemethy

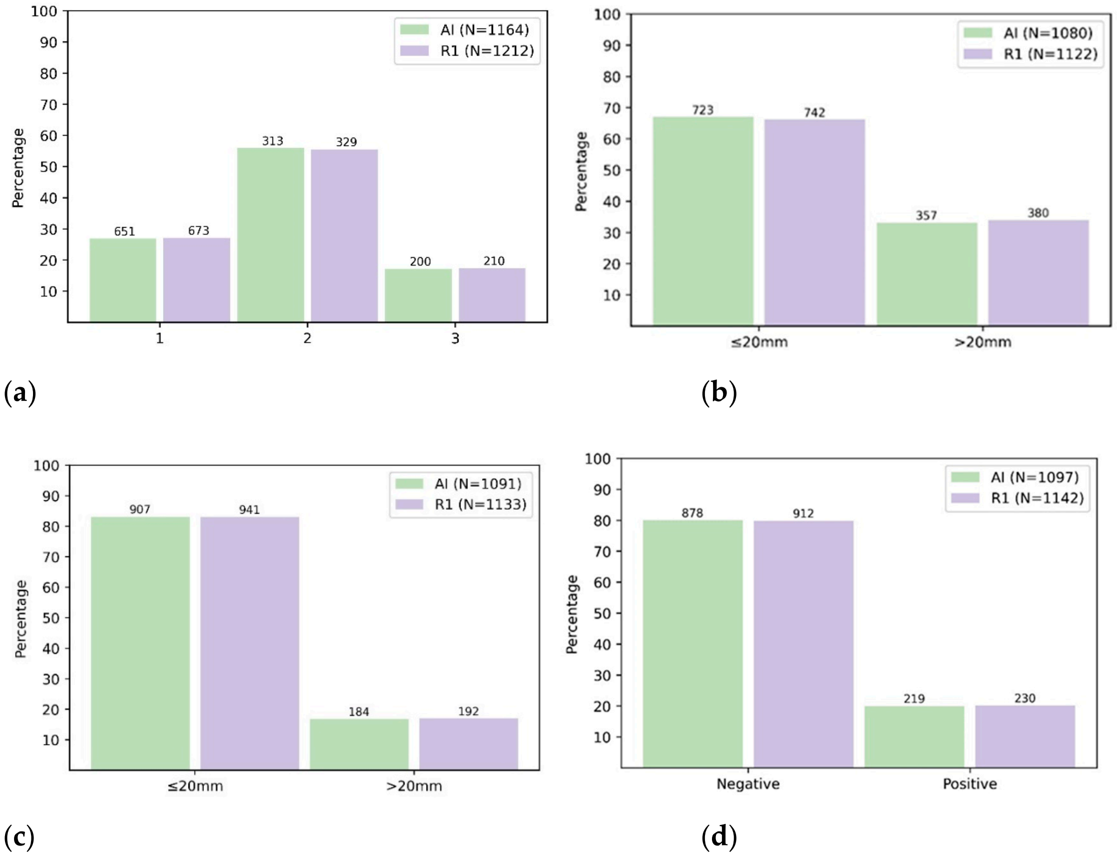

Figure S1. (a) Barplot Histological grade; **(b)** Barplot whole tumour size; **(c)** Barplot invasive tumour size; **(d)** Barplot lymph node stage.

Table S1. Agreement between human reader and AI for detection of cancers

	Screen-detected cancers N= 1718		Interval cancers N= 293		All cancers N= 2011	
R1 only	196	11.4%	8	2.7%	204	10.1%
AI only	111	6.5%	80	27.3%	191	9.5%
Detected by both	1364	79.4%	13	4.4%	1377	68.5%
Not detected by R1 or AI	47	2.7%	192	65.5%	239	11.9%

Abbreviations: R1 = human reader 1; AI = artificial intelligence

Table S2a. Size of invasive cancers detected on screen by human reader or AI

		detected by R1					detected by AI				
		N=1261	%	95% CI			N=1213	%	95% CI		
Whole tumour size	≤5 mm	38	3.4%	2.5%	-	4.6%	32	3.0%	2.1%	-	4.2%
	5-10 mm	205	18.3%	16.1%	-	20.6%	201	18.6%	16.4%	-	21.0%
	10-20 mm	499	44.5%	41.6%	-	47.4%	490	45.4%	42.4%	-	48.4%
	20-50 mm	332	29.6%	27.0%	-	32.3%	311	28.8%	26.2%	-	31.6%
	>50	48	4.3%	3.2%	-	5.6%	46	4.3%	3.2%	-	5.6%
	Missing	139					133				
Invasive tumour size	≤5 mm	114	10.1%	8.4%	-	12.0%	109	10.0%	8.3%	-	11.9%
	5-10 mm	308	27.2%	24.7%	-	29.8%	288	26.4%	23.9%	-	29.1%
	10-20 mm	519	45.8%	42.9%	-	48.7%	510	46.7%	43.8%	-	49.7%
	20-50 mm	180	15.9%	13.9%	-	18.1%	174	15.9%	13.9%	-	18.2%
	>50	12	1.1%	0.6%	-	1.8%	10	0.9%	0.5%	-	1.7%
	Missing	128					122				

Abbreviations: R1 = Human reader 1, AI = artificial intelligence

Table S2b. Size of invasive interval cancers detected by human reader or AI

		detected by R1					detected by AI				
		N=15	%	95% CI			N=65	%	95% CI		
Whole tumour size	≤5 mm	0	0.0%				2	4.7%	1.3%	-	15.5%
	5-10 mm	0	0.0%				2	4.7%	1.3%	-	15.5%
	10-20 mm	3	37.5%	13.7%	-	69.4%	15	34.9%	22.4%	-	49.8%
	20-50 mm	5	62.5%	30.6%	-	86.3%	20	46.5%	32.5%	-	61.1%
	>50	0	0.0%	0.0%	-	32.4%	4	9.3%	3.7%	-	21.6%
	Missing	7					22				
Invasive tumour size	≤5 mm	1	7.1%	1.3%	-	31.5%	4	6.7%	2.6%	-	15.9%
	5-10 mm	3	21.4%	7.6%	-	47.6%	5	8.3%	3.6%	-	18.1%
	10-20 mm	5	35.7%	16.3%	-	61.2%	25	41.7%	30.1%	-	54.3%
	20-50 mm	5	35.7%	16.3%	-	61.2%	22	36.7%	25.6%	-	49.3%
	>50	0	0.0%				4	6.7%	2.6%	-	15.9%
	Missing	1					5				

Abbreviations: R1 = Human reader 1, AI = artificial intelligence

Table S3a. Sensitivity per prognostic subgroup for screen-detected cancers

Variable		N	Detected AI	SEN AI	95% CI	Detected R1	SEN R1	95% CI	P
Invasive Component	not present	326	258	79.1%	74.8% - 83.5%	293	89.9%	86.5% - 93.1%	<0.001
	present	1385	1213	87.6%	85.8% - 89.3%	1261	91.0%	89.5% - 92.6%	0.002
	missing	7							
Tumour grade	grade 1	371	313	84.4%	80.6% - 87.9%	329	88.7%	85.3% - 91.8%	0.089
	grade 2	738	651	88.2%	85.8% - 90.5%	673	91.2%	89.1% - 93.2%	0.059
	grade 3	224	200	89.3%	85.2% - 93.1%	210	93.8%	90.5% - 96.8%	0.099
	missing	52							
Whole tumour size	≤20 mm	831	723	87.0%	84.7% - 89.3%	742	89.3%	87.1% - 91.3%	0.148
	>20 mm	408	357	87.5%	84.2% - 90.7%	380	93.1%	90.7% - 95.5%	0.006
	missing	146							
Invasive tumour size	≤20 mm	1045	907	86.8%	84.7% - 88.8%	941	90.0%	88.2% - 91.8%	0.017
	>20 mm	205	184	89.8%	85.3% - 93.9%	192	93.7%	90.3% - 96.9%	0.185
	missing	135							
Lymph node status	negative	1017	878	86.3%	84.1% - 88.4%	912	89.7%	87.7% - 91.5%	0.018
	positive	243	219	90.1%	86.3% - 93.8%	230	94.7%	91.7% - 97.4%	0.071
	missing	125							

Abbreviations: AI = artificial intelligence; R1 = human reader 1; CI = confidence interval; SEN = sensitivity

Tumour grade, whole tumour size, invasive tumour size and lymph node status were only assessed for cancers that had an invasive component present

P values are based on McNemar's test

Table S3b. Sensitivity per prognostic subgroup for interval cancers

Variable		N	Detected AI	SEN AI	95% CI			Detected R1	SEN R1	95% CI			P
Invasive Component	not present	15	6	40.0%	14.3%	-	66.7%	1	6.7%	0.0%	-	23.1%	0.0625
	present	222	65	29.3%	23.5%	-	35.3%	15	6.8%	3.6%	-	10.2%	<0.001
	missing	56											
Tumour grade	grade 1	30	16	53.3%	35.5%	-	70.8%	3	10.0%	0.0%	-	22.2%	<0.001
	grade 2	107	25	23.4%	15.4%	-	31.6%	7	6.5%	2.1%	-	11.7%	<0.001
	grade 3	83	23	27.7%	18.2%	-	37.1%	5	6.0%	1.3%	-	11.6%	<0.001
	missing	2											
Whole tumour size	≤20	80	19	23.8%	14.8%	-	33.3%	3	3.8%	0.0%	-	8.5%	<0.001
	>20	84	24	28.6%	18.9%	-	38.5%	5	6.0%	1.2%	-	11.5%	<0.001
	missing	58											
Invasive tumour size	≤20	123	34	27.6%	20.0%	-	35.8%	9	7.3%	3.0%	-	12.6%	<0.001
	>20	84	26	31.0%	21.2%	-	41.1%	5	6.0%	1.3%	-	11.5%	<0.001
	missing	15											
Lymph node status	negative	93	28	30.1%	20.9%	-	39.8%	6	6.5%	2.1%	-	12.0%	<0.001
	positive	55	16	29.1%	16.9%	-	42.1%	4	7.3%	1.5%	-	14.6%	<0.001
	missing	74											

Abbreviations: AI = artificial intelligence; R1 = human reader 1; CI = confidence interval; SEN = sensitivity

Tumour grade, whole tumour size, invasive tumour size and lymph node status were only assessed for cancers that had an invasive component present

P values are based on McNemar's test

Table S4. Relative Sensitivity of AI and R1 per subgroup for screen-detected and interval cancers combined

Variable		N	Total Detected R1	Detected AI*	Rel SEN AI	95% CI	Total Detected AI	Detected R1**	Rel SEN R1	95% CI
Invasive Component	present	1607	1276	1129	88.5%	86.7% - 90.2%	1278	1129	88.3%	86.5% - 90.1%
	not present	341	294	242	82.3%	77.8% - 86.5%	264	242	91.7%	88.2% - 94.9%
	missing	63								
Tumour grade	grade 1	401	332	284	85.5%	81.8% - 89.3%	329	284	86.3%	82.7% - 89.9%
	grade 2	845	680	604	88.8%	86.4% - 91.2%	676	604	89.3%	87.0% - 91.6%
	grade 3	307	215	194	90.2%	86.0% - 93.9%	223	194	87.0%	82.5% - 91.2%
	missing	54								
Whole tumour size	≤20 mm	911	745	656	88.1%	85.7% - 90.3%	742	656	88.4%	86.0% - 90.6%
	>20 mm	492	385	338	87.8%	84.4% - 91.0%	381	338	88.7%	85.5% - 91.6%
	missing	204								
Invasive tumour size	≤20 mm	1168	950	833	87.7%	85.6% - 89.7%	941	833	88.5%	86.5% - 90.5%
	>20 mm	289	197	178	90.4%	86.0% - 94.3%	210	178	84.8%	80.1% - 89.5%
	missing	150								
Lymph node status	negative	1110	918	801	87.3%	85.1% - 89.4%	906	801	88.4%	86.3% - 90.4%
	positive	298	234	213	91.0%	87.3% - 94.4%	235	213	90.6%	86.8% - 94.4%
	missing	199								

Abbreviations: AI = artificial intelligence; R1 = human reader 1; Rel SEN = relative sensitivity; CI = confidence interval

* Detected AI is the subgroup of cancers detected by AI that is also detected by R1

** Detected R1 is the subgroup of cancers detected by R1 that is also detected by the AI
